# Evolution of Cuticular Hydrocarbons in the Hymenoptera: a Meta-Analysis

**DOI:** 10.1007/s10886-015-0631-5

**Published:** 2015-09-26

**Authors:** Ricarda Kather, Stephen J. Martin

**Affiliations:** Department of Animal and Plant Sciences, University of Sheffield, Sheffield, S10 2TN UK; School of Environment and Life Sciences, The University of Salford, Manchester, M5 4WT UK

**Keywords:** Cuticular hydrocarbons, Communication, Sociality, Spring-loaded, Hymenoptera, Gene-silencing, Sanflies, Parasitoid wasps, Aculeate wasps, Ants, Bees

## Abstract

Chemical communication is the oldest form of communication, spreading across all forms of life. In insects, cuticular hydrocarbons (CHC) function as chemical cues for the recognition of mates, species, and nest-mates in social insects. Although much is known about the function of individual hydrocarbons and their biosynthesis, a phylogenetic overview is lacking. Here, we review the CHC profiles of 241 species of Hymenoptera, one of the largest and most important insect orders, which includes the Symphyta (sawflies), the polyphyletic Parasitica (parasitoid wasps), and the Aculeata (wasps, bees, and ants). We investigated whether these taxonomic groups differed in the presence and absence of CHC classes and whether the sociality of a species (solitarily *vs*. social) had an effect on CHC profile complexity. We found that the main CHC classes (*i.e*., *n*-alkanes, alkenes, and methylalkanes) were all present early in the evolutionary history of the Hymenoptera, as evidenced by their presence in ancient Symphyta and primitive Parasitica wasps. Throughout all groups within the Hymenoptera, the more complex a CHC the fewer species that produce it, which may reflect the Occam’s razor principle that insects’ only biosynthesize the most simple compound that fulfil its needs. Surprisingly, there was no difference in the complexity of CHC profiles between social and solitary species, with some of the most complex CHC profiles belonging to the Parasitica. This profile complexity has been maintained in the ants, but some specialization in biosynthetic pathways has led to a simplification of profiles in the aculeate wasps and bees. The absence of CHC classes in some taxa or species may be due to gene silencing or down-regulation rather than gene loss, as demonstrated by sister species having highly divergent CHC profiles, and cannot be predicted by their phylogenetic history. The presence of highly complex CHC profiles prior to the vast radiation of the social Hymenoptera indicates a ‘spring-loaded’ system where the diversity of CHC needed for the complex communication systems of social insects were already present for natural selection to act upon, rather than having evolved independently. This diversity may have aided the multiple independent evolution of sociality within the Aculeata.

**Electronic supplementary material:**

The online version of this article (doi:10.1007/s10886-015-0631-5) contains supplementary material, which is available to authorized users.

## Introduction

Chemical communication is the oldest form of communication, spreading across all forms of life (Wilson 1970), and underlies almost all known behavior from genes to super-organisms. Pheromones are one of the most important signals perceived through the chemical sensory channel (Wyatt 2013), and are particularly complex and well studied in insects (Howard and Blomquist 2005), where 1000s of pheromones have been described. Short-range contact pheromones are used by many insects to identify and potentially discriminate against other individuals of the same or different species (Wyatt 2013). The best studied group of compounds are the cuticular hydrocarbons (CHC) that are embedded in the cuticular lipid layer of all insects and have been extensively researched over the past 30 years. This has shown that CHC differ greatly both quantitatively and qualitatively among as well as within a species. More recently, CHC have been shown to convey information about an individual’s fertility, sex, gender, caste, kin, *etc*. in numerous species (Blomquist and Bagnères 2010). The majority of CHC studies have concentrated on the Hymenoptera, one of the largest and most diverse insect orders with over 130,000 described species, including many economically and environmentally important species, especially among the social bees, wasps and ants (Wilson 1971). The combined hymenopteran biomass outweighs that of all other terrestrial organisms, even the vertebrates, due to their evolutionary success, which is reflected in their vast abundance (Wilson 1971). Central to their success is their chemical ecology.

Within the Hymenoptera, a huge diversity of CHC is present with thousands of compounds already having been described. This diversity is generated simply by either the insertion of one or more double bonds (olefins) or one or more methyl groups (methylalkanes) at various positions along a chain of carbon atoms that typically varies from 21 to around 40 carbons in length. Very rarely do both biosynthetic pathways combine to produce methylalkenes, which are methylalkanes that also contain a double bond/s. Importantly, these small additions of a double bond or methyl group cause the molecules to bend *via* Van der Waals forces, so giving each CHC a unique conformation (shape). Furthermore, most methylalkanes contain chiral centers and perception depends on odorant chirality, although in 20 insect species from nine orders the methyl-branched hydrocarbons were in the (*R*)-configuration (Bello et al. 2015). Likewise the vast majority of insect olefins are present in the (*Z*)-configuration. It has been shown that insects detect these small differences in compound structure, *i.e*., the position, chirality, or absence of double bonds or methyl groups, so insects can distinguish between compounds of the same chain length that vary in the position of their double bond(s) (Dani et al. 2005) or methyl group(s) (Châline et al. 2005). However, little is known about the actual molecular mechanism at the basis of CHC perception (olfactory or gustatory receptors; involvement of possible carrier proteins *etc*.), with only a few studies having investigated antennal electrophysiological responses to CHC (*e.g*., Ozaki et al. 2005). So current evidence for differential perception of CHC with different moieties are based only on a limited number of behavioral bioassays using a very small number of species.

The thousands of CHC can be categorized into three main groups: 1) saturated *n*-alkanes, 2) olefins that contain one or more double bonds, and 3) methylalkanes, which contain one or more methyl groups. Just two main biosynthetic pathways underlie the production of all these CHC (Howard and Blomquist 2005; Morgan 2010). Both types of pathways involve the elongation and reduction of fatty acyl-CoAs precursors to aldehydes before oxidative decarbonylation to obtain the correct carbon chain length (Qiu et al. 2012). The production of *n*-alkanes and alkenes, involves malonyl-CoA and, in the case of alkenes, a fatty acyl-CoA desaturase inserting a double-bond into the carbon chain at a precise location; *i.e*., a Δ9 desaturase inserts a double bond in the 9th position, a Δ7 desaturase into the 7th position *etc*. In the production of methylalkanes, it is methylmalonyl-CoA that helps to insert a methyl group at various positions along the carbon chain.

There is increasing evidence that compound structure (*i.e*., presence and position of double-bonds or methyl groups) rather than chain length is the key factor when it comes to an insect’s ability to detect and learn different hydrocarbons (Châline et al. 2005; Dani et al. 2005; van Wilgenburg et al. 2010). These studies demonstrate that insects can easily discriminate between compounds bearing moieties such as double bond and methyl branches, but cannot discriminate linear alkanes. Furthermore, insects are able to learn and distinguish between compounds of the same chain length that vary in the position of their double-bond or methyl group, but are unable to discriminate between different homologs, *i.e*., compounds that share the same structure but differ in chain length (van Wilgenburg et al. 2010). Hence, we have concentrated on the divergence of CHC structural isomers among the Hymenoptera, and have omitted data on chain length in order to make the analysis of the dataset manageable.

Another factor that makes Hymenoptera a key system is that the order contains both solitary and social species. Solitary insects use CHC to identify mates of the correct species and gender (*e.g*., Bartelt et al. 2002; Böröczky et al. 2009; Steiner et al. 2006), whereas social insects use CHC to distinguish individuals of different species, castes, colonies, dominance statuses, developmental stages, kin, *etc*. (*e.g*., Bonavita-Cougourdan et al. 1987; Ferreira-Caliman et al. 2010; Martin et al. 2008a; Monnin 2006; Wagner et al. 2001). Given that social insects have a much greater level of chemical communication than solitary insects, it has long been assumed that social insects will produce a greater variety of CHC compared to solitary Hymenoptera; a hypothesis we test.

Here, we provide the first review of all hymenopteran CHC profiles published to date in a phylogenetic framework, and we investigate major taxonomic differences in the main CHC classes: *n*-alkanes, alkenes, and methylalkanes found across the order. A number of studies have provided an overview of hymenoptera CHC profiles at the genus (wasps: Khidr et al. 2013; bees: Hadley et al. 1981; ants: Martin et al. 2008b) or family level (ants: Martin and Drijfhout 2009; van Wilgenburg et al. 2011). However, a taxonomic overview of CHC profiles across the whole order is missing, and will provide a much needed wider and more insightful perspective into the long term evolution of CHC in this important group of insects.

## Methods and Materials

### Data Collection

The CHC profiles of 241 hymenopteran species (Appendix I) were collated using Web of Science® (http://thomsonreuters.com/web-of-science/), and were based on a total of 167 studies (Appendix II) published between 1982 and 2013. To standardize CHC profiles across the 165 social species, we used only worker CHC profiles since queen data were reported only in a small minority of studies. Because of the difficulty in correctly interpreting mass spectra of some hydrocarbon groups such as dimethylalkanes, there is a small chance that the data provided in Appendix III are not entirely free of errors. It does, however, reflect accurately what has been published. For each species, the presence and absence of a chemical class and associated isomers were recorded (Appendix III). In a number of studies, the threshold that classified a compound as occurring at ‘trace’ amounts was either missing or differed between studies. Therefore, we recorded all compounds reported, including those that had been detected at trace amounts. Species were split into five main taxonomic groups: the Symphyta ([sawflies] 2 species), the polyphyletic Parasitica ([parasitoid wasps] 27 species), the aculeate wasps (39 species), ants (95 species), and bees (78 species). According to Wilson (1971), the Symphyta are the most ancient of the five taxonomic groups present in our dataset, together with the primitive Parasitica. Therefore, these two groups could potentially provide a glimpse of the types of CHC that were already present in the early evolutionary history of the order. The more ‘modern’ Aculeata, were divided into aculeate wasps, ants, and bees, because these groups differ distinctly in life style (*i.e*., nest type, feeding behavior, *etc*.), and they contain all of the 164 social species.

All analyses were conducted using the statistical software R (v 2.81) or SPSS v. 20. Since our dataset contained only two species of Symphyta, these were excluded from the statistical analysis, but still are included in some of the figures to serve as a reference to the other four taxonomic groups.

### Taxonomic Occurrence of CHC Classes

To investigate any differences in the occurrence of the main CHC classes (Table 1) among the four taxonomic groups (Parasitica, aculeate wasps, ants, and bees), the number of species per group that produced a given CHC class were calculated, and the results were analyzed using a Cochran-Mantel-Haenszel (CMH) *χ*^2^- test. To ensure the results of the CMH test had not been impaired by phylogenetic bias, for example, because some genera were over-represented in the dataset skewing the results, we applied a measure of phylogenetic signal strength (*D*) as suggested by Fritz and Purvis (2010). *D* can be used to test for phylogenetic bias in a binary dataset in which some species are over-represented. Such over-representation of species can lead to statistical bias and false significance values (Freckleton et al. 2002). In our dataset, a number of genera, *e.g*., *Bombus*, *Polistes* and *Vespa*, were over-represented because they are popular model systems. Therefore, we applied *D* to each taxonomic group separately to test for intra-group phylogenetic bias that would affect the degree to which our results could be generalized across each taxonomic group.

**Table 1:**
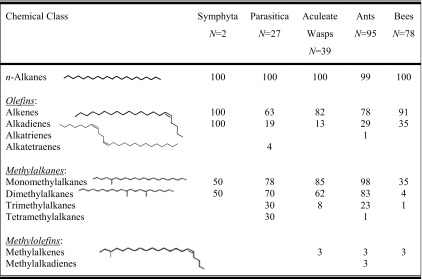
The occurrence of cuticular hydrocarbon (CHC) classes across four groups of Hymenoptera

The percentage of species in which a given chemical class was present is shown for each group. The number of species in each group (*N*) is also given

The *D* statistic is equal to 1 if the observed binary trait has a phylogenetically random distribution across the tips of the phylogeny, and is equal to 0 if the distribution is clumped. Increasing clumping in the binary trait is indicated by values of *D* decreasing from 1. If the binary trait is extremely clumped, *D* falls below 0, whereas over-dispersion of the observed binary trait is indicated by values greater than 1.

To calculate *D* we combined our binary dataset with a phylogenetic tree of the Hymenoptera, kindly provided by Peters et al. (2011) that was constructed from a supermatrix of 120,000 sequences from 1100 species. When species were present in our dataset but absent in the phylogenetic tree, we used the package ‘ape’ (Paradis et al. 2013) in R to build these missing species into the phylogenetic tree (nexus file). The UniProt database (www.uniprot.org/taxonomy/) in combination with the Animal Diversity Web (ADW) database of the University of Michigan (www.animaldiversity.ummz.umich.edu/) were used to determine the branch position of each new species within the tree. The branch length for the added species was calculated based on the average of the branch lengths of its sister-species within the same genus or (sub-) family. We used the statistical package ‘caper’ (Orme 2013) in R to estimate *D* for each CHC class (excluding the *n*-alkanes, as these were universally present throughout the Hymenoptera) across the Hymenoptera as a whole, and for each taxonomic group separately. The following very rare chemical classes (alkatrienes, alkatetraenes, tetramethylalkanes, methylalkenes, and methylalkadienes) were excluded from the above analyses because they were produced only by a handful of species and, thus, sample size was too low for these to be reliably analyzed.

To investigate the phylogenetic history on CHC production we extracted all our study species from the phylogenetic tree of the Hymenoptera (Peters et al. 2011), after it had been supplemented with our binary dataset (see above). This represents a phylogenetic tree for all our study species. The branch lengths were not shown for clarity. The final branch then was color coded depending on the class of CHC that species produced.

Finally a cladogram was produced using the binary dataset (Appendix III) of all CHC using a Hierarchical cluster analysis in SPSS v. 20, using Squared Euclidean distances with average linked (between group) functions. Again each taxonomic group was color coded to help with identifying any patterns.

### Isomer Diversity

To test for differences in positional isomer diversity across the four taxonomic groups, the average number of isomers (per CHC class) produced by each taxonomic group was compared using a generalized linear model (Poisson error structure; followed by a χ^2^test) to account for the variation in sample size between the taxonomic groups. For the rarer CHC classes (*e.g*., alkadienes, alkatrienes, alkatetraenes, tetramethylalkanes, methylalkenes, and methylalkadienes) isomer information was reported only in a small number of species and, therefore, these classes again were excluded from the analysis. When the data were too skewed to be transformed, we used a non-parametric Kruskal-Wallis test instead.

### Is Sociality Driving CHC Diversity?

To investigate differences in CHC diversity (*i.e*., the number of CHC classes present in the chemical profile, as well as the number of isomers produced) based on sociality, species were split into two groups: solitary or social species. Out of the five taxonomic groups (this time including the Symphyta), the bees and the aculeate wasps are the only two groups that contain a mixture of social and solitary life styles. The social status of each bee species was based on Michener (2007), whereas the social status of the aculeate wasps was described in the respective papers from which their CHC profile had been taken. Two generalized linear models (Poisson error structure; followed by a *χ*^2^ test) were run to test for sociality-specific differences in CHC complexity. The first analysis was run on the number of chemical classes that were produced by social and solitary species, whereas the second analysis assessed the total number of isomers (again divided by CHC class) found in the CHC profiles of the two groups. A species was included only in the latter analysis, when isomer data had been reported for all CHC classes present in that species, which resulted in a total of 40 solitary and 93 social species being included in this particular dataset.

### Biosynthetic Patterns

In the ants, a more ‘complex’ CHC (*e.g*., a compound with two methyl groups) appeared to be correlated to the compound class of the next simpler structure (*e.g*., with one methyl group), suggesting that ‘simpler’ compounds are precursors of more ‘complex’ CHC (Martin and Drijfhout 2009; Van Wilgenburg et al. 2011). Therefore, we also investigated whether and how strongly CHC classes were correlated across the Hymenoptera by applying a Kendall’s τ correlation analysis test on the presence/absence of CHC classes, as well as on the number of CHC isomers found per CHC class. Again compounds that were produced only by a handful of species (see above) were excluded from the analysis.

## Results

### Taxonomic Occurrence of CHC Classes

**Fig. 1:**
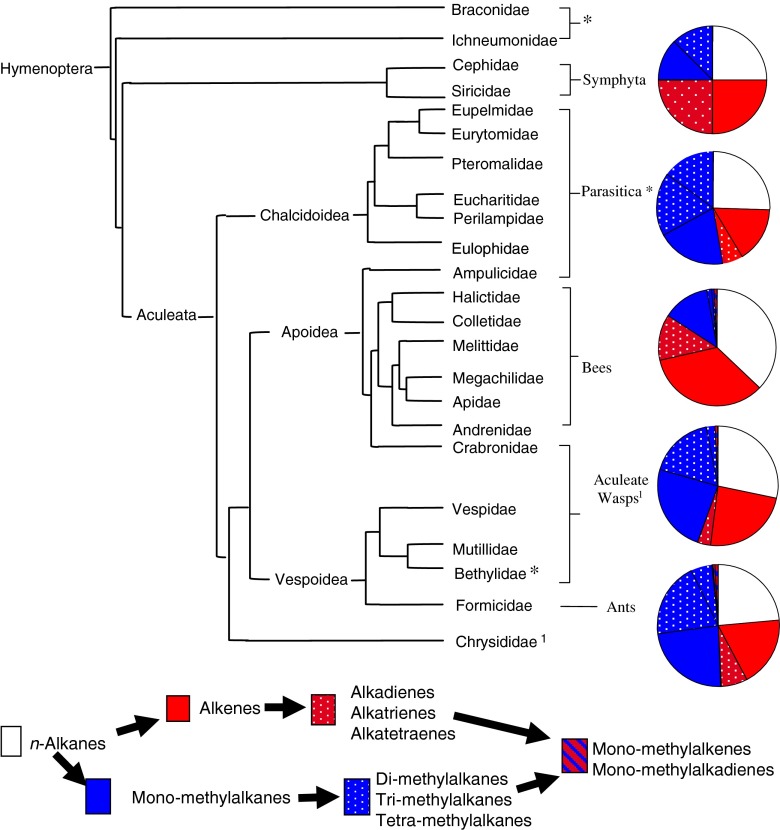
A cladogram of the Hymenoptera contained in our dataset. The phylogenetic relationships shown are based on Brothers (1999), Davis et al. (2010), and Peters et al. (2011). Hymenoptera families were split (based on their life style) into five main taxonomic groups: the Symphyta, Parasitica, aculeate wasps, ants, and bees. The Parasitica are polyphyletic and are highlighted with an asterisk (*) to show which families were classed as belonging to this group of parasitoid wasps. The same is applied to the aculeate wasps, which were marked with a number (1). The pie chart next to each taxonomic group gives an overview of the proportions of the six major cuticular hydrocarbon (CHC) class (see legend) produced by each group. The Symphyta are shown here to demonstrate that the main CHC classes found in the Hymenoptera are already present in these ‘ancient’ species of Hymenoptera. Only the bees have specialized on diversification of olefins, while the rest (ants, aculeate wasps, and parasitica) have proceeded mainly down a methyl diversification path. As our dataset contained only two species of Symphyta, the pie chart is unlikely to be a representation of the Symphyta

**Fig. 2:**
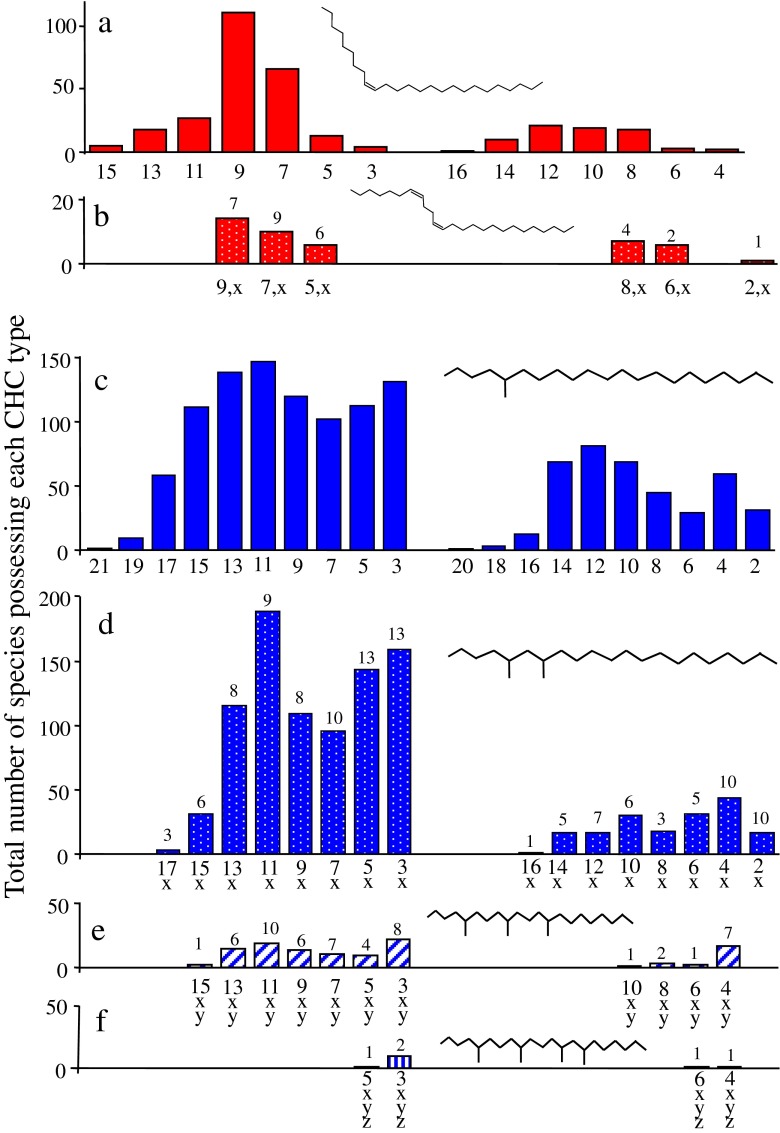
Abundance of positional isomers **a**) alkenes, **b**) dienes, **c**) monomethylalkanes, **d**) dimethylalkanes, **e**) trimethylalkanes, and **f**) tetramethylalkanes, produced by the Hymenoptera surveyed in this study. The numbers above the bars indicate the number of positional isomers that group together (*i.e.*, three dimethylalkanes such as 9,15; 9,17; and 9,19, would result in a 3 above the 9, x bar). The figure illustrates some universal properties of hymenopteran cuticular hydrocarbons (CHC), such as odd positions are always more common than even positional isomers, the number of compounds reduced with compound complexity, except in the vast radiation of the dimethylalkanes, although there are strong similarities in the patterns of abundance between the odd chained alkenes and dienes and also between the mono-, di-, and trimethylalkanes. However, these patterns are less obvious in the even chained isomers. It could be derived that the Z9- double bond is the ancestral state as it is present throughout the entire Hymenoptera as are all many monomethylalkanes. So the diversification of positional isomers had already occurred in the most ancestral species and has been maintained throughout the entire order

The published CHC profiles of the 241 species of Hymenoptera contained 11 CHC classes (Fig. 1, Table 1) and 237 different structural isomers, which could be further classified into 14 alkene, 29 alkadiene, 20 monomethylalkane, 116 dimethylalkane, 53 trimethylalkane, and 5 tetramethylalkane isomers (Fig. 2). Since each structural isomer normally occurs as a homolog series (*i.e*., at several carbon chain-lengths), the estimated number of CHC identified within the Hymenoptera will exceed 2000.

**Fig. 3:**
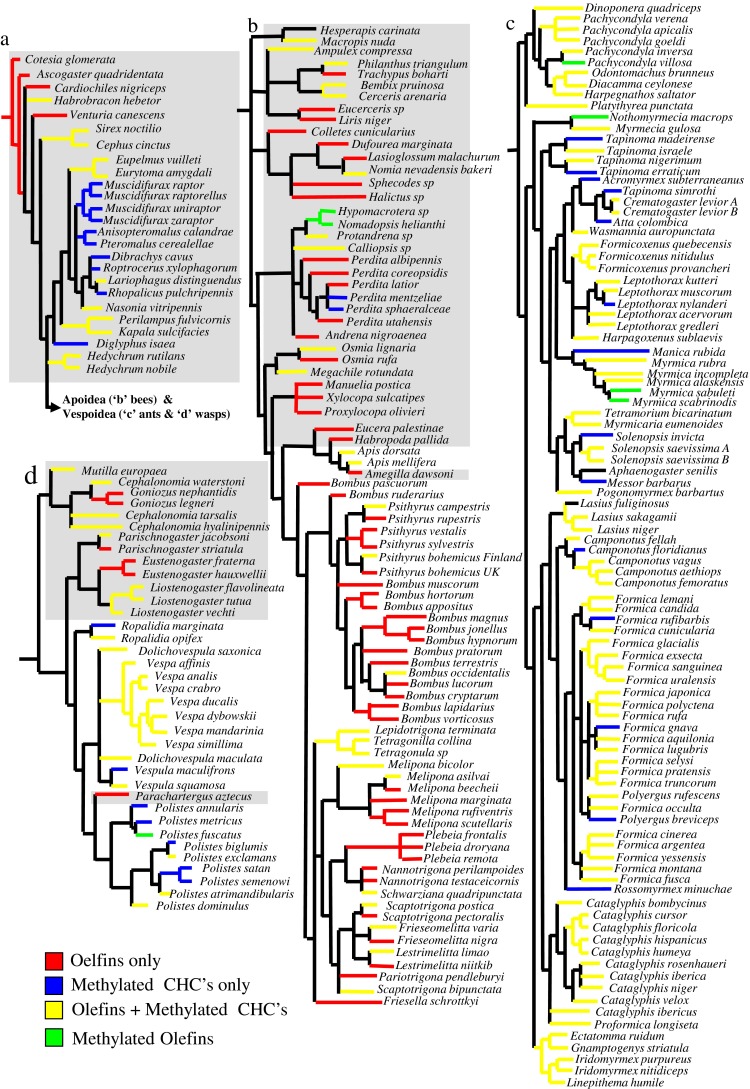
Phylogenetic tree based on 238 of the study species arranged with the each color indicating which of the four major classes of cuticular hydrocarbon (CHC) is produced by that species (the alkane only species has been excluded). The phylogenetic relationships are based on the tree by Peters et al. (2011), but the branch lengths have been ignored for the purpose of clarity. Each sub-tree represents a taxonomic group **a**) Symphyta + Parasitica; **b**) bees; **c**) ants, and **d**) aculeate wasps. The grey background indicates the solitarily species. This figure indicates specialization of olefins in the bees and methylated compounds in the wasps

All major olefin and methylalkane classes were already present in the CHC profiles of the basal Symphyta and Parasitica (Figs. 1 and 3). It appears that the olefin biochemical pathway may have preceded the methyl pathway in the Hymenoptera (see Fig. 3), even though this is based on a very small number of species. However, in these ancient groups, we already have species specializing in either olefin or methyl production, while the majority exploit both pathways. Surprisingly, the diversity among the Parasitica, which produced all but three chemical classes (alkatrienes, methylalkenes, and methylalkadienes), was greater only in the ants, as these produced all chemical classes bar one (tetraenes). The CHC profiles from Parasitica were unusual in that they contained a great diversity of tetramethylalkanes (absent from the other four taxonomic groups, including the Symphyta).

**Fig. 4:**
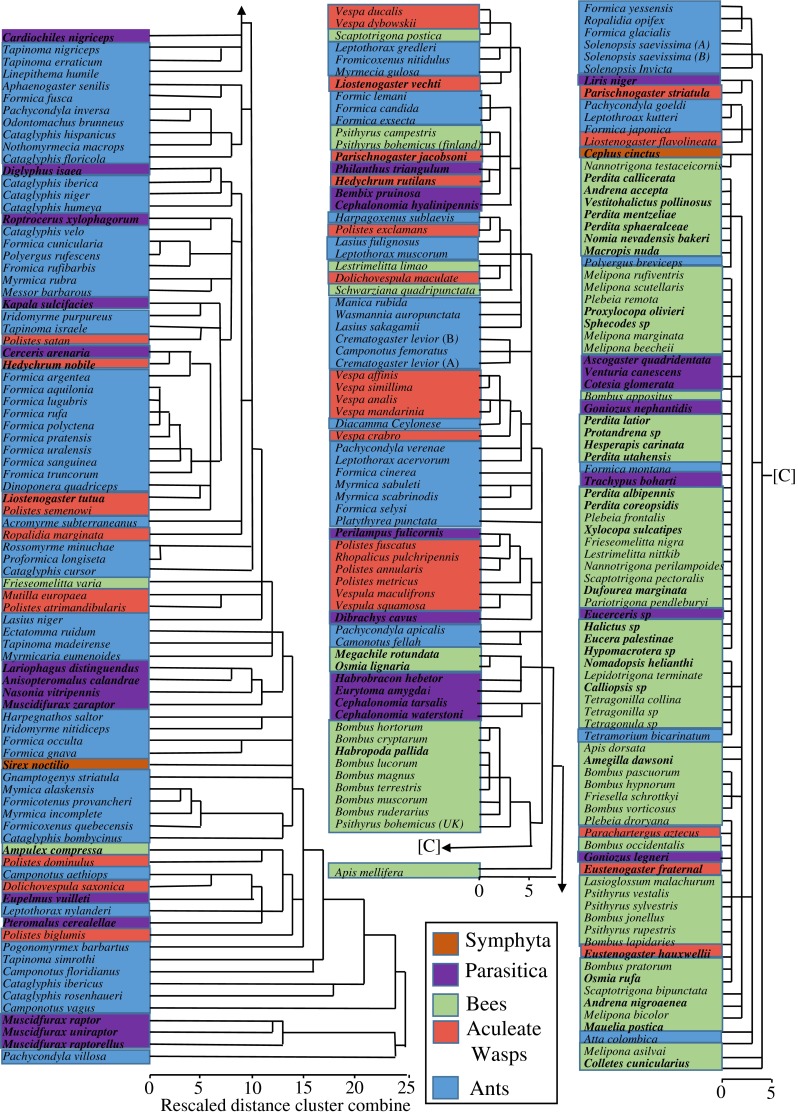
A cladogram constructed from the binary dataset of all cuticular hydrocarbons (CHC). This indicates no clear phylogenetic signal as all five taxonomic groups are dispersed throughout the entire cladogram, although the diversity of dimethylalkanes in the ants and olefins in the bees can be seen. All names of solitarily species are given in bold and again can be found throughout the cladogram indicating no clear phylogenetic chemical signal associated with the evolution of sociality

There was a significant difference in the presence and absence of alkenes (CMH test: CMH = 16.81, *d.f*. = 1, *P* < 0.001), monomethylalkanes (CMH test: CMH = 117.024, *d.f*. = 1, *P* < 0.001), dimethylalkanes (CMH test: CMH = 172.12, *d.f*. = 1, *P* < 0.001), and trimethylalkanes (CMH test: CMH = 45.91, *d.f*. = 1, *P* < 0.001) between the Parasitica, wasps, ants, and bees. This was confirmed by the *D* statistic applied to the whole order, which indicated that the presence of CHC classes was clumped particularly with regards to the alkenes (*D* = 0.48, *P* < 0.001) and the methylalkanes (monomethylalkanes: *D* = 0.19; dimethylalkanes: *D* = 0.03; trimethylalkanes: *D* = 0.39: *P* < 0.001 for all three). This clumping of CHC classes across the hymenopteran tree occurred because some genera/families specialized in producing olefins (62 species) or methylalkanes (38 species) (Figs. 3 and 4; Table 2), whereas other species used a mixed hydrocarbon approach, producing a combination of olefins and methylalkanes (124 species), as well as methylolefins (8 species) (Fig. 3).

**Table 2:** Types of cuticular hydrocarbon (CHC) profiles found among the Hymenoptera

CHC Class	*n*-Alkanes 3 species	Olefins 62 species		Methylalkanes 38 species				Olefins + Methylalkanes 124 species								Methylolefins 8 species					Exceptions 6 species					
*n*-Alkanes	X	X	X	X	X	X	X	X	X	X	X	X	X	X	X	X	X	X	X	X	X		X	X	X	X
*Olefins*:																										
Alkenes		X	X					X	X	X	X	X	X	X	X	X	X	X	X	X					X	X
Alkadienes			X								X	X	X	X	X					X			X	X	X	X
Alkatrienes															X											
Alkatetraenes																									X	
*Methylalkanes*:																										
Monomethylalkanes				X	X	X	X	X	X	X	X	X	X	X	X	X	X	X	X	X		X	X	X	X	X
Dimethylalkanes					X	X	X		X	X		X	X	X	X	X	X	X	X			X	X	X	X	
Trimethylalkanes						X	X			X			X	X		X					X			X		X
Tetramethylalkanes							X							X											X	
*Methylolefins*:																										
Methylalkenes																X	X	X		X						
Methylalkadienes																	X	X								

CHC profiles, in terms of the presence and absence of CHC classes, could be divided into five main types: Species that only produced 1) *n*-alkanes (*n*-Alkanes-only species), 2) olefins (Olefins-only species), or 3) methylalkanes (Methylalkanes-only species), and species that produced 4) a mixture of olefins and methylalkanes (Olefins + Methylalkanes-only species), as well as those that produced 5) methylolefins (Methylolefins species). Within each profile type, sub-types are shown to demonstrate the co-occurrence of ‘complex’ compounds (*i.e*., compounds with more than one double-bond or methyl group) and ‘simpler’ compounds (*i.e*., compounds with one less double-bond or methyl group). Exceptions to this pattern of correlation are shown in the final column. The number of hymenopteran species that express a given profile type are also given in the first row of the table

**Fig. 5:**
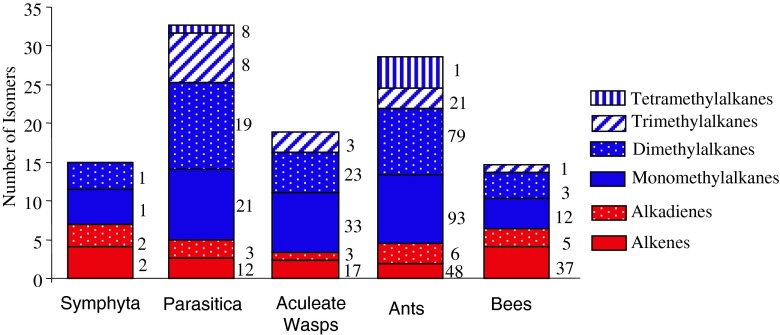
Average number of isomers across the five hymenopteran groups (Symphyta, Parasitica, aculeate wasps, ants, and bees). The number of species (per chemical class) that the isomer analysis was based on is provided next to the bars. The Symphyta are shown as a reference to the other four taxonomic groups, but were not included in the data analysis due to their low sample size

This specialization in a family or class of CHC differed among the taxonomic groups, even though there was some intra-group variation in the presence and absence of a CHC class; even within a single genus (Fig. 5). The CHC profiles of bees were dominated by olefins (62 % species) (Fig. 3), and generally lacked methylalkanes (especially dimethylalkanes and trimethylalkanes) compared to the other three taxonomic groups (Fig. 3; Table 1). Most species of aculeate wasps (64 %) and ants (72 %), however, adopted a mixed-hydrocarbon approach by producing both olefins and methylalkanes (Fig. 3). As mentioned, the Parasitica mainly produced methylalkanes-only profiles (55 % species). This general specialization is seen in the CHC cladogram (Fig. 4), as are the many exceptions to these general rules.

When the *D* statistic was repeated for each taxonomic group to test whether the presence or absence of CHC classes per group was skewed by some genera being over-represented, no such phylogenetic bias was found, which means that the patterns described above can be generalized across all species of that respective taxonomic group.

### Isomer Diversity

The four taxonomic groups differed in the number of monomethylalkane (Generalized linear model: *χ*^2^ = 40.34, *d.f*. = 3, *P* < 0.001), dimethylalkane (Kruskal-Wallis Test: *χ*^2^ = 8.75 *d.f*. = 3, *P* = 0.033) and alkene (Kruskal-Wallis Test: *χ*^2^ = 21.40, *d.f*. = 3, *P* < 0.001) isomers (Fig. 5). However, the difference in alkene isomers was driven only by the difference between ants and bees, as all other two-way comparisons were not significant (Multiple comparison after Kruskal-Wallis) (Fig. 4). Furthermore, when bees produced monomethylalkanes, they produced fewer dimethylalkanes compared to the other three taxonomic groups (*P* < 0.02), whereas the Parasitica and ants produced higher numbers of dimethylalkanes compared to the aculeate wasps and bees (*P* < 0.05). The highest number of trimethylalkanes was produced by the Parasitica (*P* < 0.001).

### Is Sociality Driving CHC Diversity?

Surprisingly, sociality had no effect on the number of chemical classes (Generalized linear model: χ = 2.66, *d.f*. = 1, *P* = 0.1), nor the number of CHC isomers (Generalized linear model: χ = 0.38, *d.f*. = 1, *P* = 0.54) produced by a species. CHC profiles associated with social species consisted of a similar number of CHC classes (4 ± 1), as well as an almost identical number of isomers (14 ± 11) compared to solitary species (average number of CHC classes: 3 ± 1; average number of CHC isomers: 15 ± 15). This is further supported by the finding that no relationship between taxonomic cladogram (Fig. 3) nor the CHC cladogram (Fig. 4) could be observed. Isomer number did not consider different carbon chain lengths.

### Biosynthetic Patterns

The more structurally complex (*i.e*., increasing number of double-bonds or methyl groups) a CHC was, the fewer species produced it (Table 1). For example, the most basic CHC are the *n*-alkanes, and they are almost universally produced among the Hymenoptera (99.6 %), whereas the structurally complex methylolefins are produced by only three species in any group. This property occurred universally across all taxonomic groups (Table 1). That is a chemical class with high structural complexity and was almost always present in conjunction with the chemical class of the next lower structural complexity (Fig. 1; Table 2). It was supported by the correlation analysis, which showed that the olefins (in this case alkenes and alkadienes) were positively correlated (Kendall’s τ for CHC class: τ = 0.457, z = 3.87, *P* < 0.001; Kendall’s τ for CHC isomers: τ = 0.25, z = 3.68, *P* < 0.001), and so were the methylalkanes (Kendall’s τ: τ > 0.2, *P* < 0.001). Olefins and methylalkanes were negatively correlated (*P* < 0.01), because many species that produced olefins did not produce methylalkanes (*e.g*., many bees), whereas species that produced many methylalkanes (*e.g*., many species of Parasitica and ants) often had fewer olefins in their CHC profiles (as described above).

## Discussion

### Evolution of CHC Classes

Our review revealed distinct differences in the CHC profiles found among the Hymenoptera. Both the ancient Symphyta and the primitive Parasitica produced almost all types of olefins and methylalkanes, which suggest that the majority of CHC classes and their associated biochemical pathways were already present early in Hymenoptera evolutionary history. Such pre-existence of the major biosynthetic pathways could explain the great diversity of CHC compounds found within the Hymenoptera, because it is likely that sister-species were able to evolve new structural isomers based on small changes in the biosynthetic pathways (Baker 2002; Symonds and Elgar 2008). The rare methylolefins were present only in the Aculeata, which indicates that the ability to combine biosynthetic pathways to produce these CHC evolved later in the Hymenoptera. However, the ability to produce methylolefins is found in the primitive ant species (*Nothomyrmecia macrops*) (Brown et al. 1990) and *Pachycondyla villosa* (D’Ettorre et al. 2005; Lucas et al. 2004), indicating that this trait was probably present prior to the evolution of bees and wasps, since these rare CHC also are produced by two solitarily bees and one social wasp species (Fig. 3). Methylolefins may be under-represented in the literature, since the interpretation of their spectra is more complicated; both because double bond positions cannot be determined from electronic ionization spectra, and because little information about the fragmentation of these molecules is currently available.

The largest diversity both with respect to number of chemical classes (Table 1) and CHC isomers (Fig. 5) were present in the primitive Parasitica and younger Formicidae (ants), both of which produce almost all CHC classes found in the Hymenoptera, indicating no loss of ability to produce the various CHC over evolutionary time. A simplification in CHC profiles was seen in the aculeate wasps, which only produced monomethylalkanes and very few dimethylalkanes and trimethylalkanes compared with their ant sister-family, which have specialized in dimethylalkane production (Figs. 4 and 5; Appendix III; Martin and Drijfhout 2009; Martin et al. 2008c) as have the Parasitica. Likewise, the bees have diversified olefin production (Figs. 4 and 5; Martin et al. 2010) and down-regulation of the methylalkane pathway over time in these clades. However, it appears that no group has lost the ability to produce any class of CHC despite apparent diversification into certain CHC groups. Even at the species-level the down-regulation of individual compounds or whole chemical classes is not uncommon. Intra-species variation in the occurrence of CHC classes has been recorded for several species of bees. For example in *Apis mellifera*, the CHC profile of the brood contains monomethylalkanes that are then almost absent at the adult stage (Kather et al. 2015; Nation et al. 1992). Such dimorphism in methylalkane production also has been observed when comparing the CHC of queens and their (sister) workers, for example in *Melipona bicolor* (Abdalla et al. 2003) and *Friesella schrottkyi* (Nunes et al. 2010). In both these species, queens produce methylalkanes even though these are absent or occur at very low concentrations in the workers. In the parasitic wasp, *Lariophagus distinguendus*, males deactivate the production of 3-methylheptacosane 32 h after emergence so that they are no longer mistaken for females (Steiner et al. 2007). Such evidence of intra-species CHC variation suggests that entire groups of genes may be silenced or down-regulated rather than lost completely, as is often assumed. The silencing of genes over very long periods of evolutionary time helps explain the intra-genus variation in the presence and absence of certain CHC classes that was observed in several genera within our dataset (*e.g*., in the bee genus *Perdita*, the aculeate wasp genus *Polistes*, and the ant genus *Formica*) (Fig. 3). Despite this, throughout the Hymenoptera there seems to exist the universal rule that the more complex a CHC the fewer the species produce it. This may reflect the Occam’s razor principle that insect’s only biosynthesize the simplest CHC to perform the required task and only produce more complex compounds when under strong selection pressure to do so.

As expected, diversification of a particular group of CHC in the bees (olefins) or Parasitica and aculeate wasps (methylalkanes) is reflected in their role in communication. For example, nest mate recognition is associated with alkenes in *Trigona fulviventris* (Buchwald and Breed 2005) and *Apis mellifera* (Dani et al. 2005; Pradella et al. 2015), while in other bees, alkenes serve as sex pheromones and initiate mating in *Colletes cunicularis* (Mant et al. 2005), *Andrena nigroaenea* (Schiestl et al. 1999), *Habropoda pallida* (Saul-Gershenz and Millar 2006), and *Megachile rotundata* (Paulmier et al. 1999). In *Frieseomelitta varia*, olefins appear to act as queen pheromones (Nunes et al. 2009). Whereas, in the Parasitica, such as *Nasonia vitripennis* (Steiner et al. 2007), *Eupelmus vuilleti* (Darrouzet et al. 2010), *Dibrachys cavus* (Ruther et al. 2011), *Roptrocerus xylophagorum* (Sullivan 2002) and *Lariophagus distinguendus* (Steiner et al. 2007), mono- and dimethylalkanes act as short-range sex pheromones. In the aculeate wasps (*Polistes*, *Ropalidia* and *Vespa*), monomethylalkanes are linked to nest mate recognition (Dani et al. 1996; Dapporto et al. 2006; Espelie et al. 1994; Layton et al. 1994; Lorenzi et al. 1997, 2004; Ruther et al. 2002; Tannure-Nascimento et al. 2007). In the few wasp species where methylalkanes were absent but olefins present, such as *Cardiochiles nigripes* (Syvertsen et al. 1995) and *Cephus cinctus* (Bartelt et al. 2002), sexes of these species differ in their alkene and diene quantities, and these may function as a contact sex pheromones.

Trying to use lifestyle to explain the major diversification of certain CHC groups, such as olefin diversification in the bees, currently fails to produce a coherent story. Social wasps and bees both live and forage in the same environment, but have diversified in methylalkane and olefin production, respectively. However, one possible avenue of further research is that the production of methylalkanes requires the use of essential amino acids such as valine and methionine. These amino acids can be obtained only *via* the ingestion of proteinaceous foods such as meat in wasps and ants. Bees obtain their essential amino acids by feeding on pollen. Therefore, there could be a relationship between diet, in respect to the availability of protein and methylalkane production.

### Is Sociality Driving CHC Diversity?

Our analysis rejected the long standing assumption that CHC complexity is linked to sociality, since no sociality-based differences in CHC profiles were found. In fact, surprisingly both the solitary Parasitica and the social ants produced the most complex CHC profiles across the Hymenoptera. Many other solitary insects such as flies (Diptera) can have relatively simple CHC profiles (Carlson and Yocom 2005; Ferveur 2005), although exceptions do exist (Nelson et al. 1981). Parasitica are known to have complex courtship behaviors, in which chemical signals play a greater role than visual or tactile cues (Sullivan 2002). In many parasitoid species, CHC play a crucial part in locating, recognizing and assessing potential mates (Ayasse et al. 2001; Johansson and Jones 2007; Matthews 1975; Singer 1998; Sullivan 2002). They also facilitate the coordination of courtship behavior (Ruther et al. 2011; Steiner et al. 2006). Intense host competition also has led to parasitoids using CHC to mark hosts to reduce intra-host competition (Van Alphen and Visser 1990). Such factors could contribute to the selection of complex CHC profiles. Whatever the reason, this study reveals that the high CHC diversity required by all social species was already present prior to the evolution of sociality. Therefore, the primitive Parasitica represent a “spring-loaded” system (Nowak et al. 2010). This is where the ability to produce a diverse range of CHC needed for the evolution of a communication system as complex as that used by social insects, was already present for natural selection to act upon, rather than having to evolve it independently. This is evidenced by the 225 odorant receptors (Ors) present in the solitary *Nasonia* wasp (Robertson et al. 2010), relative to the 174 Ors present in honeybees, both which greatly exceed the number found in flies *Drosophila melanogaster* (62 Ors) and *Anopheles gambiae* (79 Ors) (Robertson and Wanner 2006). This type of spring-loaded pre-adaptation may be a key factor in aiding the evolution of sociality in many different groups within the Aculeata.

## Electronic supplementary material

ESM 1 (XLS 876 kb)

ESM 2 (PDF 43 kb)

ESM 3 (PDF 87.1 kb)
