# Supplementary material for: Evolution of Cuticular Hydrocarbons in the Hymenoptera: a Meta-Analysis
Source: J Chem Ecol. 2015 Sep 26;41(10):871–83. doi: 10.1007/s10886-015-0631-5 (PMC4619461; doi:10.1007/s10886-015-0631-5)
Supplement: Supplementary file 2 — (PDF 43 kb) [file 10886_2015_631_MOESM2_ESM.pdf]

Appendix I A summary of the 241 hymenopteran species that were contained in our dataset. Species are grouped according to the taxonomic family and genus they belong to. Column 1 states which one of the five taxonomic groups that species were allocated to, and it follows the order of the cladogram shown in Fig. 1. The reference(s) that the CHC profile of a species was based on is provided in column 5 and a full reference list of all 170 studies is given in Appendix II.

Taxonomic group	Family	Genus	Species	References
Parasitica	Braconidae	Cotesia	*Cotesia glomerata*	Ruf et al. (2010)
		Ascogaster	*Ascogaster quadridentata*	Espelie and Brown (1990)
		Cardiochiles	*Cardiochiles nigriceps*	Syvertesen et al. (1995)
		Venturia	*Habrobracon hebetor*	Howard and Baker (2003a)
	Ichneumonidae	Habrobracon	*Venturia canescens*	Marris et al. (1996)
Symphyta	Cephidae	Sirex	*Cephus cinctus*	Bartelt et al. (2002)
	Siricidae	Cephus	*Sirex noctilio*	Boroczky et al. (2009)
Parasitica	Eupelmidae	Eupelmus	*Eupelmus vuilleti*	Darrouzet et al. (2010)
	Eurytomidae	Eurytoma	*Eurytoma amygdali*	Krokos et al. (2001)
	Pteromalidae	Dibrachys	*Dibrachys_cavus*	Ruther et al. (2011)
		Lariophagus	*Lariophagus distinguendus*	Steiner et al. (2007)
				Kuhbandner et al. (2012)
		Pteromalus	*Pteromalus cerealellae*	Howard (2001)
		Muscidifurax	*Muscidifurax uniraptor*	Bernier et al. (1998)
			Muscidifurax raptor	Bernier et al. (1998)
			Muscidifurax zaraptor	Bernier et al. (1998)
			Muscidifurax raptorellus	Bernier et al. (1998)
		Nasiona	*Nasonia vitripennis*	Steiner et al. (2006)
		Anisopteromalus	*Anisopteromalus calandrae*	Howard and Baker (2003b)
		Roptrocerus	*Roptrocerus xylophagorum*	Sullivan (2002)
		Rhopalicus	*Rhopalicus pulchripennis*	Espelie et al. (1990)
	Eucharitidae	Kapala	*Kapala sulcifacies*	Howard et al. (2001)
	Perilampidae	Diglyphus	*Perilampus fulvicornis*	Espelie and Brown (1990)
	Eulophidae	Perilampus	*Diglyphus isaea*	Finidori-Logli et al. (1996)
	Ampulicidae	Ampulex	*Ampulex_compressa*	Herzner et al. 2011
Acueate wasps	Crabronidae	Trachypus	*Trachypus boharti*	Koedam et al. (2011)
		Liris	*Liris niger*	Gnatzy et al. (2004)
		Bembix	*Bembix pruinosa*	Hadley et al. (1981)
		Eucerceris	*Eucerceris sp.*	Clarke et al. (2001)
		Cerceris	*Cerceris arenaria*	Strohm et al. (2008b)
		Philanthus	*Philanthus triangulum*	Strohm et al. (2008)
				Kroiss et al. (2009)
Bees	Melittidae	Macropis	*Macropis nuda*	Cane (1983)
		Hesperapis	*Hesperapis carinata*	Cane (1983)
	Colletidae	Colletes	*Colletes cunicularius*	Mant et al. (2005)
				Vereecken et al. (2007)
	Halictidae	Sphecodes	*Sphecodes sp*	Tengo et al. (1992)
				Sick et al. (1994)
		Halictus	*Halictus sp*	Hefetz et al. (1986)
				Hefetz and Graur (1988)
				Sick et al. (1994)
			Vestitohalictus pollinosus	Hefetz and Graur (1988)
		Lasioglossum	*Lasioglossum malachurum*	Hefetz et al. (1986)
				Sick et al. (1994)
				Ayasse et al. (1999)
				Soro et al. (2011)
		Nomia	*Nomia nevadensis bakeri*	Hadley et al. (1981)
		Dufourea	*Dufourea marginata*	Cane (1983)
	Megachilidae	Megachile	*Megachile rotundata*	Paulmier et al. (1999)
				Buckner et al. (2009)
		Osmia	*Osmia lignaria*	Guedot et al. (2006)
				Buckner et al. (2009)
	Apidae	Eucera	*Eucera palestinae Friese*	Shimron et al. (1985)
		Habropoda	*Habropoda pallida*	Saul-Gershenz and Millar (2006)
		Amegilla	*Amegilla dawsoni*	Simmons et al. (2003)
		Apis	*Apis mellifera*	Blomquist et al. (1980)
				Dani et al. 2004
				Strohm et al. (2008)
				del Piccolo et al. (2010)
				Carlson et al. (1989)
			Apis dorsata	Carlson et al. (1991)

Appendix I Table 1 (continued).

Taxonomic group	Family	Genus	Species	References
Bees	Apidae	Bombus	*Bombus appositus*	Hadley et al. (1981)
			Bombus occidentalis	Hadley et al. (1981)
			Bombus lapidarius	Tengo et al. (1991)
				Oldham et al. (1994)
				Martin et al. (2010)
			Bombus hortorum	Martin et al. (2010)
			Bombus muscorum	Martin et al. (2010)
			Bombus jonellus	Martin et al. (2010)
			Bombus lucorum	Tengo et al. (1991)
				Martin et al. (2010)
			Bombus pascuorum	Oldham et al. (1994)
				Martin et al. (2010)
				Tengo et al. (1991)
			Bombus hypnorum	Tengo et al. (1991)
				Hefetz et al. (1993)
			Bombus cryptarum	Tengo et al. (1991)
			Bombus magnus	Tengo et al. (1991)
			Bombus ruderarius	Tengo et al. (1991)
			Bombus pratorum	Tengo et al. (1991)
				Oldham et al. (1994)
			Bombus vorticosus	Tengo et al. (1991)
			Bombus (Psithyrus) campestris	Martin et al. (2010)
			Bombus (Psithyrus) rupestris	Martin et al. (2010)
			Bombus (Psithyrus) vestalis	Martin et al. (2010)
			Bombus (Psithyrus) sylvestris	Martin et al. (2010)
			Bombus (Psithyrus) bohemicus	Martin et al. (2010)
Bees	Apidae	Tetragonilla	*Tetragonilla collina*	Leonhardt et al. (2009)
		Tetragonula	*Tetragonula sp.*	Leonhardt et al. (2009)
		Pariotrigona	*Pariotrigona pendleburyi*	Leonhardt et al. (2009)
		Lepidotrigona	*Lepidotrigona terminata*	Leonhardt et al. (2009)
		Nannotrigona	*Nannotrigona testaceicornis*	Pianaro et al. (2009)
			Nannotrigona perilampoides	Quezada-Euan et al. (2013)
		Melipona	*Melipona asilvai*	Nascimento and Nascimento (2012)
			Melipona beecheii	Quezada-Euan et al. (2013)
			Melipona bicolor	Abdalla et al. (2003)
			Melipona marginata	Ferreira-Caliman et al. (2010)
			Melipona scutellaris	Kerr et al. (2004)
				Pianaro et al. (2007)
			Melipona rufiventris	Pianaro et al. (2007)
		Scaptotrigona	*Scaptotrigona bipunctata*	Jungnickel et al. (2004)
			Scaptotrigona postica	Koedam et al. (2011)
			Scaptotrigona pectoralis	Quezada-Euan et al. (2013)
		Frieseomelitta	*Frieseomelitta varia*	Nunes et al. (2008)
				Nunes et al. (2009b)
			Frieseomelitta nigra	Quezada-Euan et al. (2013)
		Friesella	*Friesella schrottkyi*	Nunes et al. (2010)
		Lestrimelitta	*Lestrimelitta limao*	Nunes et al. (2008)
			Lestrimelitta niitkib	Quezada-Euan et al. (2013)
		Schwarziana	*Schwarziana quadripunctata*	Nunes et al. (2009a)
		Plebeia	*Plebeia frontalis*	Quezada-Euan et al. (2013)
			Plebeia remota	Francisco et al. (2008)
			Plebeia droryana	Pianaro et al. (2009)
		Xylocopa	*Xylocopa sulcatipes*	Kronenberg and Hefetz (1984)
			Proxylocopa olivieri	Kronenberg and Hefetz (1984)
		Manuelia	*Manuelia postica*	Flores-Prado et al. (2008)
	Andrenidae	Calliopsis	*Calliopsis sp.*	Hefetz et al. (1982)
				Cane (1983)
			Hypomacrotera sp.	Cane (1983)
			Nomadopsis helianthi	Cane (1983)
		Perdita	*Perdita albipennis*	Cane (1983)
			Perdita coreopsidis	Cane (1983)

Appendix I Table 1 (continued).

Taxonomic group	Family	Genus	Species	References
Bees	Apidae	Bombus	*Bombus terrestris*	Tengo et al. (1991)
				Oldham et al. (1994)
				Amsalem et al. (2009)
				Martin et al. (2010)
		Perdita	*Perdita utahensis*	Cane (1983)
			Perdita callicerata	Cane (1983)
			Perdita mentzeliae	Cane (1983)
			Perdita sphaeralceae	Cane (1983)
			Perdita latior	Cane (1983)
		Pseudopanurg	*Pseudopanurgus aethiops*	Cane (1983)
		Protandrena	*Protandrena sp.*	Cane (1983)
		Andrena	*Andrena accepta*	Cane (1983)
			Andrena nigroaenea	Schiestl and Ayasse (2000)
Ants	Formicidae	Dinoponera	*Dinoponera quadriceps*	Monnin et al. (1998)
		Pachycondyla	*Pachycondyla apicalis*	Hefetz et al. (2001)
			Pachycondyla goeldi	Denis et al. (2006)
			Pachycondyla inversa	Tentschert et al. (2001)
			Pachycondyla villosa	Lucas et al. (2004)
				D'Ettore and Heinze (2005)
		Odontomachus	*Odontomachus brunneus*	Smith et al. (2012)
		Diacamma	*Diacamma ceylonese*	Cuvillier-Hot et al. (2001)
		Harpegnathos	*Harpegnathos saltator*	Liebig et al. (2000)
		Platythyrea	*Platythyrea punctata*	Hartmann et al. (2005)
		Pachycondyla	*Pachycondyla verenae*	Evison et al. (2012)
		Nothomyrmecia	*Nothomyrmecia macrops*	Brown et al. (1990)
		Ectatomma	*Ectatomma ruidum*	Howard et al. (2001)
		Gnamptogenys	*Gnamptogenys striatula*	Lommelen et al. (2006)
		Linepithema	*Linepithema humile*	Liang et al. (2001)
		Tapinoma	*Tapinoma madeirense*	Berville et al. (2013)
			Tapinoma israele	Berville et al. (2013)
			Tapinoma nigerimum	Berville et al. (2013)
			Tapinoma erraticum	Berville et al. (2013)
			Tapinoma simrothi	Berville et al. (2013)
		Acromyrmex	*Acromyrmex subterraneanus*	Richard et al. (2004)
		Iridomyrmex	*Iridomyrmex nitidiceps*	Brophy et al. (1983)
			Iridomyrmex purpureus	Brophy et al. (1973)
				van Wilgenburg et al. (2006)
		Myrmecia	*Myrmecia gulosa*	Dietemann et al. (2003)
		Atta	*Atta columbica*	Martin and MacConnell (1970)
		Wasmannia	*Wasmannia auropunctata*	Errard et al. (2005)
		Harpagoxenus	*Harpagoxenus sublaevis*	Kaib et al. (1993)
		Manica	*Manica rubida*	Bagnères et al. (1991)
		Myrmica	*Myrmica rubra*	Bagneres and Morgan (1990)
			Myrmica incompleta	Lenoir et al. (1997)
				Lohman et al. (2006)
			Myrmica alaskensis	Lenoir et al. (1997)
				Lohman et al. (2006)
			Myrmica sabuleti	Guillem et al. (2012)
			Myrmica scabrinodis	Guillem et al. (2012)
		Tetramorium	*Tetramorium bicarinatum*	Astruc et al. (2001)
		Myrmicaria	*Myrmicaria eumenoides*	Kaib et al. (2000)
		Solenopsis	*Solenopsis invicta*	Obin (1986)
				VanderMeer et al. (1989)
			Solenopsis saevissima A	Fox et al. (2012)
			Solenopsis saevissima B	Fox et al. (2012)
		Aphaenogaster	*Aphaenogaster senilis*	Lenoir et al. (2001)
		Messor	*Messor barbarus*	Provost et al. (1994)
		Pogonomyrmex	*Pogonomyrmex barbatus*	Wagner et al. (1998)
				Nelson et al. (2001)
		Leptothorax	*Leptothorax kutteri*	Franks et al. (1990)
			Leptothorax acervorum	Kaib et al. (1993)
				Tentschert et al. (2002)
			Leptothorax gredleri	Tentchert et al. (2002)
			Leptothorax muscorum	Kaib et al. (1993)

Appendix I Table 1 (continued).

Taxonomic group	Family	Genus	Species	References
Ants	Formicidae	Leptothorax	*Leptothorax nylanderi*	Trabalon et al. (2000)
		Formicoxenus	*Formicoxenus quebecensis*	Lenoir et al. (1997)
			Formicoxenus nitidulus	Martin et al. (2007)
			Formicoxenus provancheri	Lenoir et al. (1997)
		Crematogaster	*Crematogaster levior A*	Emery and Tsutsui (2013)
			Crematogaster levior B	Emery and Tsutsui (2013)
		Lasius	*Lasius fuliginosus*	Akino (2002)
			Lasius sakagamii	Akino and Yamaoka (1998)
			Lasius niger	Akino and Yamaoka (1998)
		Camponotus	*Camponotus aethiops*	Boulay et al. (2000)<br>Katzav-Godansky (2004)
			Camponotus fellah	Endler et al. (2004)
			Camponotus floridianus	Meskali et al. (1995)
			Camponotus vagus	Akino (2002)
			Camponotus femoratus	Emery and Tsutsui (2013)
		Rossomyrmex	*Rossomyrmex minuchae*	Errard et al. (2006)
		Proformica	*Proformica longiseta*	Errard et al. (2006)
		Formica	*Formica selysi*	Bagneres et al. (1991)
			Formica pratensis	Martin et al. (2008)
			Formica truncorum	Akino (2006)<br>Martin et al. (2008)
			Formica occulta	Johnson et al. (2001)
			Formica rufibarbis	Bonavita-Cougordon (1997, 2004)
			Formica cunicularia	Bonavita-Cougordon (1997)
			Formica montana	Henderson et al. (1990)
			Formica fusca	Martin et al. (2008)<br>Hannonen et al. (2002)
			Formica gnava	Johnson et al. (2001)
			Formica aquilonia	Martin et al. (2007)
			Formica lugubris	Martin et al. (2007)
			Formica japonica	Akino et al. (2004)
			Formica polyctena	Martin et al. (2007)
			Formica rufa	Martin et al. (2007)
			Formica glacialis	Lohman et al. (2006)
			Formica exsecta	Martin et al. (2007)
			Formica sanguinea	Martin et al. (2007)
			Formica uralensis	Martin et al. (2007)
			Formica cinerea	Martin et al. (2007)
			Formica argentea	Krasnec and Breed (2013)
			Formica yessensis	Kidokoro-Kobayashi et al. (2012)
			Formica lemani	Martin et al. (2008)
			Formica candida	Martin et al. (2008)
		Polyergus	*Polyergus breviceps*	Johnson et al. (2001)
			Polyergus rufescens	Bonavita-Cougordon (1997, 2004)
		Cataglyphis	*Cataglyphis bombycinus*	Dahbi et al. (1996)
			Cataglyphis ibericus	Dahbi et al. (1996, 2008)<br>Dabhi and Lenoir (1998)
			Cataglyphis cursor	Nowbahari et al. (1990)
			Cataglyphis floricola	Dahbi et al. (1996)
			Cataglyphis hispanicus	Dahbi et al. (1996)
			Cataglyphis humeya	Dahbi et al. (1996)
			Cataglyphis rosenhaueri	Dahbi et al. (1996)
			Cataglyphis iberica	Dahbi et al. (2008)
			Cataglyphis niger	Lahav et al. (2001)
		Cataglyphis	*Cataglyphis velox*	Dahbi et al. (1996)
Aculeate Wasps	Mutillidae	Mutilla	*Mutilla europaea*	Uboni et al. (2012)
Parasitoid	Bethylidae	Cephalonomia	*Cephalonomia hyalinipennis*	Howard and Perez-Lachaud (2002)
			Cephalonomia tarsalis	Howard and Baker (2003b)

Taxonomic group	Family	Genus	Species	References
Parasitoid	Bethylidae	Cephalonomia	*Cephalonomia waterstoni*	Howard and Baker (2003b)
		Goniozus	*Goniozus nephantidis*	Khidr et al. (2013)
			Goniozus legneri	Khidr et al. (2013)
Aculeate Wasps	Vespidae	Liostenogaster	*Liostenogaster flavolineata*	Bernier et al. (1998)
				Cervo et al. (2002)
			Liostenogaster tutua	Sledge et al. (2000)
			Liostenogaster vechti	Turillazzi et al. (2004)
		Eustenogaster	*Eustenogaster fraterna*	Sledge et al. (2000)
				Turillazzi et al. (2004)
			Eustenogaster hauxwellii	Sledge et al. (2000)
		Parischnogaster	*Parischnogaster jacobsoni*	Bernier et al. (1998)
				Sledge et al. (2000)
			Parischnogaster striatula	Zanetti et al. (2001)
		Ropalidia	*Ropalidia opifex*	Dapporto et al. (2006)
			Ropalidia marginata	Bhadra et al. 2010
		Parachartergus	*Parachartergus aztecus*	Espelie and Hermann (1988)
		Vespa	*Vespa dybowskii*	Martin et al. (2008)
			Vespa simillima	Martin et al. (2008)
			Vespa crabro	Butts et al. (1991)
				Butts et al. (1995)
				Ruther et al. (1998)
				Ruther et al. (2002)
				Spiewok et al. (2006)
				Martin et al. (2008)
			Vespa analis	Martin et al. (2008)
			Vespa mandarinia	Martin et al. (2008)
			Vespa ducalis	Martin et al. (2008)
			Vespa affinis	Martin et al. (2008)
		Dolichovespula	*Dolichovespula maculata*	Butts et al. (1991)
			Dolichovespula saxonica	Bonckaert et al. (2011)
		Vespula	*Vespula squamosa*	Butts et al. (1991)
			Vespula maculifrons	Butts et al. (1991)
		Polistes	*Polistes fuscatus*	Espelie et al. (1994)
				Panek et al. (2001)
			Polistes annularis	Espelie and Hermann (1990)
			Polistes metricus	Layton et al. (1994)
				Espelie et al. (1990b)
			Polistes exclamans	Singer (1992)
			Polistes dominulus	Bonavita-Cougourdan et al. (1991)
				Dani et al. (1996)
				Sledge et al. (2001)
				Dapporto et al. (2004)
				Lorenzi et al. (2004)
				Sledge et al. (2004)
			Polistes biglumis	Lorenzi et al. (1997)
			Polistes satan	Tannure-Nascimento et al. (2007)
			Polistes semenowi	Lorenzi et al. (2004)
			Polistes atrimandibularis	Uboni et al. (2012)
	Chrysididae	Hedychrum	*Hedychrum rutilans*	Strohm et al. (2008)
				Kroiss et al. (2009)
			Hedychrum nobile	Strohm et al. (2008)
