# Supplementary material for: Evolution of Cuticular Hydrocarbons in the Hymenoptera: a Meta-Analysis
Source: J Chem Ecol. 2015 Sep 26;41(10):871–83. doi: 10.1007/s10886-015-0631-5 (PMC4619461; doi:10.1007/s10886-015-0631-5)
Supplement: Supplementary file 3 — (PDF 87.1 kb) [file 10886_2015_631_MOESM3_ESM.pdf]

APPENDIX II Full Reference list of Species given in Appendix I

Abdalla FC, Jones GR, Morgan ED, da Cruz-Landin C (2003) Comparative study of the cuticular hydrocarbon composition of *Melipona bicolor* Lepeletier, 1836 (Hymenoptera, Meliponini) workers and queens. Genetic Mol Res 2:191–199

Akino T, Yamaoka R (1998) Chemical mimicry in the root aphid parasitoid *Paralipsis eikoae* Yasumatsu (Hymenoptera: Aphidiidae) of the aphid-attending ant *Lasius sakagamii* Yamauchi & Hayashida (Hymenoptera: Formicidae). Chemoecology 8:153–161.

Akino T (2002) Chemical camouflage by myrmecophilous beetles *Zyras comes* (Coleoptera: Staphylinidae) and *Diaritiger fossulatus* (Coleoptera: Pselaphidae) to be integrated into the nest of *Lasius fuliginosus* (Hymenoptera: Formicidae). Chemoecology 12:83–89.

Akino T (2004) Diet-induced chemical phytomimesis by twig-like caterpillars of *Biston robustum* Butler (Lepidoptera: Geometridae). Chemoecology 14:165–174.

Akino T (2006) Cuticular hydrocarbons of *Formica truncorum* (Hymenoptera: Formicidae): Description of new very long chained hydrocarbon components. Appl Entomol Zool 41:667–677.

Amsalem E, Twele R. Francke W, Hefetz A (2009) Reproductive competition in the bumble-bee *Bombus terrestris*: do workers advertise sterility? Proc Roy Soc B 276:1295–1304.

Astruc C, Malosse C, Errard C (2001) Lack of Intraspecific Aggression in the Ant *Tetramorium bicarinatum*: A Chemical Hypothesis. J Chem Ecol 27:1229–1248.

Ayasse M, Engels W, Lubke G, Taghizadeh T, Francke W (1999) Mating expenditures reduced via female sex pheromone modulation in the primitively eusocial halictine bee, *Lasioglossum (Evylaeus) malachurum* (Hymenoptera: Halictidae). Behav Ecol Sociobiol 45:95–106.

Bagnères AG, Morgan ED (1990) A simple method for analysis of insect cuticular hydrocarbons. J Chem Ecol 16:3263–3276.

Bagneres A.-G, Morgan ED (1991) The postpharyngeal glands and the cuticle of Formicidae contain the same characteristic hydrocarbons. Experientia 47:106-111.

Bartelt RJ, Cosse LA, Petroski RJ, Weaver DK (2002) Cuticular hydrocarbons and novel alkenediol diacetates from wheat stem sawfly (*Cephus cinctus*): Natural oxidation to pheromone components. J Chem Ecol 28:385–405.

Bernier UR, Carlson DA, Geden CJ (1998) Gas chromatography mass spectrometry analysis of the cuticular hydrocarbons from parasitic wasps of the genus *Muscidifurax*. J Am Soc Mass Spectr 9:320–332.

Berville L (2013) Differentiation of the ant genus *Tapinoma* (Hymenoptera: Formicidae) from the Mediterranean Basin by species-specific cuticular hydrocarbon profiles. Myrmecological News 18:77–92.

Bhadra A et al (2010) Regulation of Reproduction in the Primitively Eusocial Wasp *Ropalidia marginata*: on the Trail of the Queen Pheromone. J Chem Ecol 36:424–431.

Blomquist GJ et al (1980) Biosynthesis of wax in the honeybee, *Apis mellifera* L. Insect Biochem 10:313–321.

Bonavita-Cougourdan A et al (1991) Cuticular hydrocarbons, social organization and ovarian development in a polistine wasp *Polistes dominulus* Christ. Comp Biochem Phys B 100:667–680.

Bonavita-Cougourdan A, bagneres A-G, Provost E, Dusticier G, Clement J-L (1997) Plasticity of the cuticular hydrocarbon profile of the slave-making ant *Polyergus rufescens* depending on the social environment. Comp Biochem and Phys B 116:287-302.

Bonavita-Cougourdan A, Provost E, Riviere G, Bagneres A-G, Dusticier G (2004) Regulation of cuticular and postpharyngeal hydrocarbons in the slave-making ant *Polyergus rufescens*: effect of *Formica rufibarbis* slaves. J Insect Physiol 50:285–293.

Bonckaert W, VanZweden JS, D'Ettorre P, Billen J, Wenseleers T (2011) Colony stage and not facultative policing explains pattern of worker reproduction in the Saxon wasp. Mol Ecol 20:3455–3468.

Böröczky K et al (2009) Monoalkenes as Contact Sex Pheromone Components of the Woodwasp *Sirex noctilio*. J Chem Ecol 35:1202–1211.

Bos N, Grinsted L, Holman L (2011) Wax On, Wax Off: Nest Soil Facilitates Indirect Transfer of Recognition Cues between Ant Nestmates. PLoS ONE 6, e19435.

Boulay R, Soroker V, Godzinska EJ, Hefetz A, Lenoir A (2000) *Camponotus fellah* colony integration: worker individuality necessitates frequent hydrocarbon exchanges. Anim Behav 59:1127–1133.

Brophy JJ, Cavill GWK, Shannon JS (1973) Venom and Dufour's gland secretions in an Australian species of *Camponotus*. J Insect Physiol 19:791–798.

Brophy JJ et al (1983) Hydrocarbon constituents of three species of dolichoderine ants. Insect Biochem 13:381–389.

Brown WV, et al (1990) Preliminary examination of cuticular hydrocarbons of worker termites as chemotaxonomic characters for some Australian species of *Coptotermes* (Isoptera, Rhinotermitidae). Sociobiology 16:305–328.

Buckner JS et al (2009) Cuticular lipids of female solitary bees, *Osmia lignaria* Say and *Megachile rotundata* (F.) (Hymenoptera: Megachilidae). Comp Biochem Physiol B 153:200–205.

Butts DP, Espelie KE, Hermann HR (1991) Cuticular hydrocarbons of 4 species of social wasps in the subfamily Vespinae, *Vespa crabro* L., *Dolichovespula maculata* (L), *Vespula squamosa* (Drury), and *Vespula maculifrons* (Buysson). Comp Biochem Physiol B 99:87–91.

Butts DP, Camann MA, Espelie KE (1995) Workers and queens of the European hornet *Vespa crabro* L have colony-specific cuticular hydrocarbon profiles (Hymenoptera, Vespidae). Insect Soc 42:45–55.

Cane JH (1983) Preliminary chemosystematics of the Andrenidae and exocrine lipid evolution of the short-tongued bees (Hymenoptera, Apoidea). Syst Zool 32:417–430.

Carlson DA, Roan C-S, Yost, RA, Hector J (1989) Dimethyl disulfide derivatives of long-chain alkenes, alkadienes, and alkatrienes for Gas-Chromatography Mass-Spectrometry. Anal Chem 61:1564–1571.

Carlson DA, Roubik DW, Milstrey K (1991) Distinctive hydrocarbons among giant honey bees, the *Apis dorsata* group (Hymenoptera, Apidae). Apidologie 22:169–181.

Cervo R, Dani FR, Zanetti P, Massolo A, Turillazzi S (2002) Chemical nestmate recognition in a stenogastrine wasp, *Liostenogaster flavolineata* (Hymenoptera Vespidae). Ethol Ecol Evol 14:351–363.

Clarke SR, Dani FR, Jones GR, Morgan ED, Schmidt JO (2001) (Z)-3-Hexenyl (R)-3-hydroxybutanoate: A male specific compound in three North American decorator wasps *Eucerceris rubripes, E. conata* and *E. tricolor*. J Chem Ecol 27:1437–1447.

Taina Conrad RJP (2010) Female choice in the red mason bee, *Osmia rufa* (L.) (Megachilidae). J Exp Biol 213:4065–73.

Cuvillier-Hot V, Cobb M, Malosse C, Peeters C (2001) Sex, age and ovarian activity affect cuticular hydrocarbons in *Diacamma ceylonense*, a queenless ant. J Insect Physiol 47:485–493.

Dahbi A, Lenoir A (1998) Nest separation and the dynamics of the Gestalt odour in the polydomous ant *Cataglyphis iberica* (Hymenoptera, Formicidae). Behav Ecol Sociobiol 42:349–355.

Dahbi A et al (1996) Chemistry of the postpharyngeal gland secretion and its implication for the phylogeny of Iberian *Cataglyphis species* (Hymenoptera: Formicidae). Chemoecology 7:163–171.

Dahbi A, Hefetz A, Lenoir A (2008) Chemotaxonomy of some Cataglyphis ants from Morocco and Burkina Faso. Biochem Syst Ecol 36:564–572.

Dani FR, Morgan ED, Turillazzi S (1996) Dufour gland secretion of Polistes wasp: Chemical composition and possible involvement in nestmate recognition (Hymenoptera: vespidae). J Insect Physiol 42:541–548.

Dani FR, Corsi S, Pradella D, Jones GR, Turillazzi S (2004) GC-MS analysis of epicuticle lipids of *Apis mellifera* reared in central Italy. Insect Social Life 5:103–109.

Dapporto L, Theodora P, Spacchini C, Pieraccini G, Turillazzi S (2004) Rank and epicuticular hydrocarbons in different populations of the paper wasp *Polistes dominulus* (Christ) (Hymenoptera, Vespidae). Insect Soc 51:279–286.

Dapporto L, Fondelli L, Turillazzi S (2006) Nestmate recognition and identification of cuticular hydrocarbons composition in the swarm founding paper wasp *Ropalidia opifex*. Biochem Syst Ecol 34:617–625.

Darrouzet E, Lebreton S, Gouix N, Wipf A, Bagneres A-G (2010) Parasitoids Modify Their Oviposition Behavior According to the Sexual Origin of Conspecific Cuticular Hydrocarbon Traces. J Chem Ecol 36:1092–1100.

Del Piccolo F, Nazzi F, Della-Vedova G, Milani N (2010) Selection of *Apis mellifera* workers by the parasitic mite *Varroa destructor* using host cuticular hydrocarbons. Parasitology 137:967–973.

Denis D, Blatrix R, Fresneau D (2006) How an ant manages to display individual and colonial signals by using the same channel. J Chem Ecol 32:1647–1661.

D'Ettorre P, kellner K, Delabie JHC, Heinze J (2005) Number of queens in founding associations of the ponerine ant *Pachycondyla villosa*. Insect Soc 52:327-332.

Dietemann V, Peeters C, Liebig J (2003) Cuticular hydrocarbons mediate discrimination of reproductives and nonreproductives in the ant *Myrmecia gulosa*. P Natl Acad Sci-Bio 100:10341–10346.

Emery VJ, Tsutsui ND (2013) Recognition in a Social Symbiosis: Chemical Phenotypes and Nestmate Recognition Behaviors of Neotropical Parabiotic Ants. PLoS ONE 8, e56492.

Endler A et al (2004) Surface hydrocarbons of queen eggs regulate worker reproduction in a social insect. P Natl Acad Sci-Bio 101:2945–2950.

Errard C, Delabie J, Jourdan H, Hefetz A (2005) Intercontinental chemical variation in the invasive ant *Wasmannia auropunctata* (Roger) (Hymenoptera Formicidae): a key to the invasive success of a tramp species. Naturwissenschaften 92:319–323.

Errard C et al (2006) Co-evolution-driven cuticular hydrocarbon variation between the slave-making ant *Rossomyrmex minuchae* and its host *Proformica longiseta* (Hymenoptera: Formicidae). Chemoecology 16:235–240.

Espelie KE, Hermann HR (1988) Congruent cuticular hydrocarbons, biochemical convergence of a social wasp, an ant and a host plant. Biochem Syst Ecol 16:505–508.

Espelie KE, Brown JJ (1990) Cuticular hydrocarbons of species which interact on 4 trophic levels, apple, *Malus pumila* Miss; codling moth, *Cydia pomonella* L.; a Hymenopteran parasitoid, *Ascogaster quadridentata* Wesmael; and a hyperparasite, *Perilampus fulvicornis* Ashmead. Comp Biochem Physiol B 95:131–136.

Espelie KE, Berisford CW, Dahlsten DL (1990a) Cuticular hydrocarbons of geographically isolated populations of *Rhopalicus pulchripennis* (Hymenoptera, Pteromalidae). Evidence for 2 species. Comp Biochem Physiol B 96:305–308.

Espelie KE, Wenzel JW, Chang G (1990b) Surface lipids of the social wasp *Polistes annularis* (L) and its nest and nest pedicel. J Chem Ecol 16:1841–1852.

Espelie K, Chapman RF, Sword GA (1994) Variation in the surface lipids of the grasshopper, *Schistocerca americana* (Drury). Biochem Syst Ecol 22:563–575.

Evison SEF, Ferreira RS, D'Ettorre P, Fresneau D, Poteaux C (2012) Chemical Signature and Reproductive Status in the Facultatively Polygynous ant *Pachycondyla Verenae*. J Chem Ecol 38:1441–1449.

Ferreira-Caliman MJ, et al (2010) The cuticular hydrocarbons profiles in the stingless bee *Melipona marginata* reflect task-related differences. J Insect Physiol 56:800–804.

Finidori-Logli V, Bagnères A-G,Erdmann D, Francke W, Clément J-L. (1996) Sex recognition in *Diglyphus isaea* Walker (Hymenoptera: Eulophidae): Role of an uncommon family of behaviorally active compounds. J Chem Ecol 22:2063–2079.

Flores-Prado L, Aguilera-Olivares D, Niemeyer HM (2008) Nest-mate recognition in *Manuelia postica* (Apidae: Xylocopinae): an eusocial trait is present in a solitary bee. Proc Roy Soc B 275:285–291.

Fox EGP et al (2012) Intraspecific and Intracolonial Variation in the Profile of Venom Alkaloids and Cuticular Hydrocarbons of the Fire Ant *Solenopsis saevissima* Smith (Hymenoptera: Formicidae). Psyche, Article ID 398061.

Francisco FO et al (2008) Morphometrical, biochemical and molecular tools for assessing biodiversity. An example in *Plebeia remota* (Holmberg, 1903) (Apidae, Meliponini). Insect Soc 55:231–237.

Franks N, Blum M, Smith RK, Allies AB (1990) Behavior and chemical disguise of cuckoo ant *Leptothorax kutteri* in relation to its host *Leptothorax acervorum*. J Chem Ecol 16:1431–1444.

Gnatzy W, Volknandt W, Schulz, S (2004) Dufour gland of the digger wasp *Liris niger*: structure and developmental and biochemical aspects. Cell and Tissue Res 315:125–138.

Guedot C, Pitts-Singer TL, Buckner JS, Bosch J, Kemp WP (2006) Olfactory cues and nest recognition in the solitary bee *Osmia lignaria*. Physiol Entomol 31:110–119.

Guillem RM, Drijfhout F, Martin SJ (2012) Using chemo-taxonomy of host ants to help conserve the large blue butterfly. Biol Conserv 148:39-43.

Hadley NF, Blomquist GJ, Lanham UN (1981) Cuticular hydrocarbons of four species of Colorado hymenoptera. Insect Biochem 11:173–177.

Hannonen H, Matthew M, Sledge F, Turillazzi S, Sundström L (2002) Queen reproduction, chemical signalling and worker behaviour in polygyne colonies of the ant *Formica fusca.* Animal Behav 64:477–485.

Hartmann A, D'Ettorre P, Jones GR, Heinze J (2005) Fertility signaling—the proximate mechanism of worker policing in a clonal ant. Naturwissenschaften 92:282–286.

Hefetz A, Graur D. (1988) The significance of multicomponent pheromones in denoting specific compositions. Biochem Syst Ecol 16:557–566.

Hefetz A, Eickwort GC, Blum MS, Cane J, Bohart GE (1982) A comparative study of the exocrine products of cleptoparasitic bees (*Holcopasites*) and their hosts (*Calliopsis)* (Hymenoptera, Anthophoridae, Andrendidae). J Chem Ecol 8:1389–1397.

Hefetz A, Bergström G, Tengö J (1986) Species, individual and kin specific blends in Dufour's gland secretions of halictine bees. J Chem Ecol 12:197–208.

Hefetz A, Tengö J, Lübke G, Francke W (1993) Inter-colonial and intra-colonial variation in Dufour's gland secretion in the bumble bee species *Bombus hypnorum* (Hymenoptera: Apidae). In: K. Wiese, F.G. Gribakin, A.V. Popov and G. Renninger (Eds.) Sensory Systems of Arthropods. Basel, Birkhäuser Verlag. pp. 469–480.

Hefetz A, Soroker V, Dahbi A, Malherbe MC, Fresneau D (2001) The front basitarsal brush in *Pachycondyla apicalis* and its role in hydrocarbon circulation. Chemoecology 11:17–24.

Henderson G, Andersen JF, Phillips JK, Jeanne RL (1990) Internest aggression and identification of possible nestmate discrimination pheromones in polygynous ant *Formica montana*. J Chem Ecol 16:2217–2228.

Herzner G et al (2011) Structure, chemical composition and putative function of the postpharyngeal gland of the emerald cockroach wasp, *Ampulex compressa* (Hymenoptera, Ampulicidae). Zoology 114:36–45.

Howard RW (2001) Cuticular hydrocarbons of adult *Pteromalus cerealellae* (Hymenoptera: Pteromalidae) and two larval hosts, angoumois grain moth (Lepidoptera: Gelechiidae) and cowpea weevil (Coleptera: Bruchidae). Ann Entomol Soc Am 94:152–158.

Howard RW, Perez-Lachaud G (2002) Cuticular hydrocarbons of the ectoparasitic wasp *Cephalonomia hyalinipennis* (Hymenoptera: Bethylidae) and its alternative host, the stored product pest *Caulophilus oryzae* (Coleoptera: Curculionidae). Arch Insect Biochem 50:75–84.

Howard RW, Baker JE (2003a) Cuticular hydrocarbons and wax esters of the ectoparasitoid *Habrobracon hebetor*: Ontogenetic, reproductive, and nutritional effects. Arch Insect Biochem 53:1–18.

Howard RW, Baker JE (2003b) Morphology and chemistry of Dufour glands in four ectoparasitoids: *Cephalonomia tarsalis*, *C. waterstoni* (Hymenoptera: Bethylidae), *Anisopteromalus calandrae*, and *Pteromalus cerealellae* (Hymenoptera: Pteromalidae). Comp Biochem Physiol B 135:153–167.

Howard RW, Pérez-Lachaud G, Lachaud J-P (2001) Cuticular hydrocarbons of *Kapala sulcifacies* (Hymenoptera: Eucharitidae) and its host, the ponerine ant *Ectatomma ruidum* (Hymenoptera: Formicidae). Ann Entomol Soc Am 94:707–716.

Johnson CA, Vander-Meer RK, Lavine B (2001) Changes in the Cuticular Hydrocarbon Profile of the Slave-Maker Ant Queen, *Polyergus breviceps* Emery, After Killing a Formica Host Queen (Hymenoptera: Formicidae). J Chem Ecol 27:1787–1804.

Jungnickel H et al (2004) Chemical basis for inter-colonial aggression in the stingless bee *Scaptotrigona bipunctata* (Hymenoptera: Apidae). J Insect Physiol 50:761–766.

Kaib M, Heinze J, Ortius D (1993) Cuticular hydrocarbon profiles in the slave-making ant *Harpagoxenus sublaevis* and its hosts. Naturwissenschaften 80:281–285.

Kaib M et al (2000) Task-related variation of postpharyngeal and cuticular hydrocarbon compositions in the ant *Myrmicaria eumenoides*. J Comp Physiol A 186:939–948.

Katzav-Gozansky T, Boulay R, Soroker V, Hefetz A (2004) Queen-signal modulation of worker pheromonal composition in honeybees. Proc Roy Soc B 271:2065–2069.

Kerr WE, Jungnickel H, Morgan ED (2004) Workers of the stingless bee *Melipona scutellaris* are more similar to males than to queens in their cuticular compounds. Apidologie 35:611–618.

Khidr SK, Linforth RST, Hardy ICW (2013) Genetic and environmental influences on the cuticular hydrocarbon profiles of Goniozus wasps. Entomol Exp Appl 147:175–185.

Kidokoro-Kobayashi M et al (2012) Chemical Discrimination and Aggressiveness via Cuticular Hydrocarbons in a Supercolony-Forming Ant, *Formica yessensis*. PLoS One 7, e46840.

Koedam D, Morgan ED, Nunes TM, Patricio EFLR, Fonseca VLI (2011) Selective preying of the sphecid wasp *Trachypus boharti* on the meliponine bee *Scaptotrigona postica*: potential involvement of caste-specific cuticular hydrocarbons. Physiol Entomol 36:187–193.

Krasnec MO, Breed MD (2013) Colony-Specific Cuticular Hydrocarbon Profile in *Formica argentea* Ants. J Chem Ecol 39:59–66.

Kroiss J, Schmitt T, Strohn E (2009) Low level of cuticular hydrocarbons in a parasitoid of a solitary digger wasp and its potential for concealment. Entomol Science 12:9–16.

Krokos FD, Konstantopoulou MA, Mazomenos BE (2001) Alkadienes and alkenes, sex pheromone components of the almond seed wasp *Eurytoma amygdali*. J Chem Ecol 27:2169–2181.

Kronenberg S, Hefetz A (1984) Comparative analysis of dufour gland secretions of 2 carpenter bees (Xylocopinae, Anthophoridae) with different nesting habits. Comp Biochem Physiol B 79:421–425.

Kühbandner S, Hacker K, Niedermayer S,. Steidle JLM, Ruther J (2012) Composition of cuticular lipids in the pteromalid wasp *Lariophagus distinguendus* is host dependent. Bull Entomol Res 102:610–617.

Lahav S, Soroker V, Vander-Meer RK, Hefetz A (2001) Segregation of Colony Odor in the Desert Ant *Cataglyphis niger*. J Chem Ecol 27:927–943.

Layton JM, Camann MA, Espelie KE (1994) Cuticular lipid profiles of queens, workers, and males of social wasp *Polistes metricus* Say are colony-specific. J Chem Ecol 20:2307–2321.

Lenoir A, Malosse C, Yamaoka R (1997) Chemical mimicry between parasitic ants of the genus Formicoxenus and their host Myrmica (Hymenoptera, Formicidae). Biochem Syst Ecol 25:379–389.

Lenoir A, Cuisset D, Hefetz A (2001) Effects of social isolation on hydrocarbon pattern and nestmate recognition in the ant *Aphaenogaster senilis* (Hymenoptera, Formicidae). Insect Soc 48:101–109.

Leonhardt SD, Bluthgen N, Schmitt T (2009) Smelling like resin: terpenoids account for species-specific cuticular profiles in Southeast-Asian stingless bees. Insect Soc 56:157–170.

Liang D, Blomquist GJ, Silverman J (2001) Hydrocarbon-released nestmate aggression in the Argentine ant, *Linepithema humile*, following encounters with insect prey. Comp Biochem Physiol B 129:871–882.

Liebig J, Peeters C, Oldham NJ, Markstädter C, Hölldobler B (2000) Are variations in cuticular hydrocarbons of queens and workers a reliable signal of fertility in the ant *Harpegnathos saltator*? P Natl Acad Sci-Bio 97:4124–4131.

Lohman DJ, Liao Q, Pierce NE (2006) Convergence of chemical mimicry in a guild of aphid predators. Ecol Entomol 31:41–51.

Lommelen E et al (2006) Cuticular hydrocarbons provide reliable cues of fertility in the ant *Gnamptogenys striatula*. J Chem Ecol 32:2023–2034.

Lorenzi MC, Bagneres A-G, Clément JL, Turillazzi S (1997) *Polistes biglumis bimaculatus* epicuticular hydrocarbons and nestmate recognition (Hymenoptera, Vespidae). Insect Soc 44:123–138.

Lorenzi MC, Sledge MF, Laiolo P, Sturlini E, Turillazzi S (2004) Cuticular hydrocarbon dynamics in young adult *Polistes dominulus* (Hymenoptera: Vespidae) and the role of linear hydrocarbons in nestmate recognition systems. J Insect Physiol 50:935–941.

Lucas C, Pho DB, D Fresneau D, Jallon JM (2004) Hydrocarbon circulation and colonial signature in *Pachycondyla villosa*. J Insect Physiol 50:595–607.

Mant J et al (2005) Cuticular hydrocarbons as sex pheromone of the bee *Colletes cunicularius* and the key to its mimicry by the sexually deceptive orchid, *Ophrys exaltata*. J Chem Ecol 31:1765–1787.

Marris GC, Hubbard SF, Scrimgeour C (1996) The perception of genetic similarity by the solitary parthenogenetic parasitoid *Venturia canescens*, and its effects on the occurrence of superparasitism. Entomol Exp Appl 78:167–174.

Martin MM, MacConnell JG (1970) The alkanes of the ant, *Atta colombica*. Tetrahedron 26:307–319.

Martin SJ, Jenner EA, Drijfhout FP (2007) Chemical deterrent enables a socially parasitic ant to invade multiple hosts. Proc Roy Soc B 274:2717–2722.

Martin SJ, Helanterä H, Drijfhout FP (2008a) Evolution of species-specific cuticular hydrocarbon patterns in Formica ants. Biol J Linn Soc 95:131–140.

Martin SJ, Takahashi J, Ono M, Drijfhout FP (2008b) Is the social parasite *Vespa dybowskii* using chemical transparency to get her eggs accepted? J Insect Physiol 54:700–707.

Martin SJ, Carruthers JM, Williams PH, Drijfhout, FP (2010) Host specific social parasites (Psithyrus) indicate chemical recognition system in bumblebees. J Chem Ecol 36:855–63.

Meskali M et al (1995) Mechanism underlying cuticular hydrocarbon homogeneity in the ant *Camponotus vagus* (SCOP.) (Hymenoptera: Formicidae): Role of postpharyngeal glands. J Chem Ecol 21:1127–1148.

Monnin T (2006) Chemical recognition of reproductive status in social insects. Ann Zool Fenn 43:515–530.

Nascimento DL, Nascimento FS (2012) Acceptance Threshold Hypothesis is Supported by Chemical Similarity of Cuticular Hydrocarbons in a Stingless Bee, *Melipona asilvai*. J Chem Ecol 38:1432–1440.

Nelson LJ, Cool LG, Forschler BT, Haverty MI (2001) Correspondence of soldier defence secretion mixtures with cuticular hydrocarbon phenotypes for chemotaxonomy of the termite genus Reticulitermes in North America. J Chem Ecol 27:1449–1479.

Nowbahari E et al (1990) Individual, geographical and experimental variation of cuticular hydrocarbons of the ant *Cataglyphis cursor* (Hymenoptera: Formicidae): Their use in nest and subspecies recognition. Biochem Syst Ecol 18:63–73.

Nunes TM, Nascimento FS, Turatti IC, Lopes NP, Zucchi R (2008) Nestmate recognition in a stingless bee: does the similarity of chemical cues determine guard acceptance? Anim Behav 75:1165–1171.

Nunes TM, Turatti IC, Lopes NP, Zucchi R (2009a) Chemical Signals in the Stingless Bee, *Frieseomelitta varia*, Indicate Caste, Gender, Age, and Reproductive Status. J Chem Ecol 35:1172–1180.

Nunes TM et al (2009b) Cuticular hydrocarbons in the stingless bee *Schwarziana quadripunctata* (Hymenoptera, Apidae, Meliponini): differences between colonies, castes and age. Genet Mol Res 8:589–595.

Nunes TM, Morgan ED, Drijfhout FP, Zucchi R (2010) Caste-specific cuticular lipids in the stingless bee *Friesella schrottkyi*. Apidologie 41:579–588.

Obin MS (1986) Nestmate recognition cues in laboratory and field colonies of *Solenopsis invicta buren* (Hymenoptera: Formicidae). J Chem Ecol 12:1965–1975.

Oldham NJ, Billen J, Morgan ED (1994) On the similarity of the dufour gland secretion and the cuticular hydrocarbons of some bumblebees. Physiol Entomol 19:115–123.

Panek LM, Gamboa GJ, Espelie KE (2001) The effect of a wasp's age on its cuticular hydrocarbon profile and its tolerance by nestmate and non-nestmate conspecifics (*Polistes fuscatus*, Hymenoptera: Vespidae). Ethology 107:55–63.

Paulmier I et al (1999) Alkenes as a sexual pheromone in the alfalfa leaf-cutter bee *Megachile rotundata*. J Chem Ecol 25:471–490.

Pianaro A, Flach A, Patricio EFLRA, Nogueira-Neto P, Marsaioli AJ (2007) Chemical changes associated with the invasion of a *Melipona scutellaris* colony by *Melipona rufiventris* workers. J Chem Ecol 33:971–984.

Pianaro A et al (2009) Stingless Bees: Chemical Differences and Potential Functions in *Nannotrigona testaceicornis* and *Plebeia droryana* Males and Workers. J Chem Ecol 35:1117–1128.

Provost E, Riviere G, Roux M, Bagneres A-G, Clement JL (1994) Cuticular hydrocarbons whereby *Messor barbarus* ant workers putatively discriminate between monogynous and polygynous colonies. Are workers labeled by queens? J Chem Ecol 20:2985–3003.

Quezada-Euán JJG et al (2013) Does sensory deception matter in eusocial obligate food robber systems? A study of *Lestrimelitta* and stingless bee hosts. Anim Behav 85:817–823.

Richard F-J, Hefetz A, Christides P, Errard C (2004) Food influence on colonial recognition and chemical signature between nestmates in the fungus-growing ant *Acromyrmex subterraneus subterraneus*. Chemoecology 14:9–16.

Ruf D, Mazzi D, Dorn S (2010) No kin discrimination in female mate choice of a parasitoid with complementary sex determination. Behav Ecol 21:1301–1307.

Ruther, J. et al. (1998) Role of cuticular lipids in nestmate recognition of the European hornet *Vespa crabro* L. (Hymenoptera, Vespidae). Insect Soc 45:169–179.

Ruther J, Sieben S Schricker B (2002) Nestmate recognition in social wasps: manipulation of hydrocarbon profiles induces aggression in the European hornet. Naturwissenschaften 89:111–114.

Ruther J, Döring M, Steiner S (2011) Cuticular hydrocarbons as contact sex pheromone in the parasitoid *Dibrachys cavus*. Entomol Exp App 140:59–68.

Saul-Gershenz LS, Millar JG (2006) Phoretic nest parasites use sexual deception to obtain transport to their host's nest. P Natl Acad Sci-Bio 103:14039–14044.

Schiestl FP, Ayasse M (2000) Post-mating odor in females of the solitary bee, *Andrena nigroaenea* (Apoidea, Andrenidae), inhibits male mating behaviour. Behav Ecol Sociobiol 48:303–307.

Shimron O, Hefetz A, Tengo J (1985) Structural and communicative functions of Dufours gland secretion in *Eucera palestinae* (Hymenoptera, Anthophoridae). Insect Biochem 15:635–638.

Sick M et al (1994) Host-parasite relationships in 6 species of Sphecodes bees and their Halictic hosts. Nest intrusion, intranidal behavior, and dufours gland volatiles (Hymenoptera, Halictidae). J Insect Behav 7:101–117.

Simmons LW, Alcock J, Reeder A (2003) The role of cuticular hydrocarbons in male attraction and repulsion by female Dawson's burrowing bee, *Amegilla dawsoni*. Anim Behav 66:677–685.

Singer TL, Camann MA, Espelie KE (1992) Discriminant analysis of cuticular hydrocarbons of social wasp *Polistes exclamans* Viereck and surface hydrocarbons of its nest paper and pedicel. J Chem Ecol 18:785–797.

Sledge MF et al (2000) Use of Dufour's gland secretion in nest defence and brood nutrition by hover wasps (Hymenoptera, Stenogastrinae). J Insect Physiol 46:753–761.

Sledge MF, Boscaro F, Turillazzi S (2001) Cuticular hydrocarbons and reproductive status in the social wasp *Polistes dominulus*. Behav Ecol Sociobiol 49:401–409.

Sledge MF, Trinca I, Massolo A, Boscaro F, Turillazzi S (2004) Variation in cuticular hydrocarbon signatures, hormonal correlates and establishment of reproductive dominance in a Polistine wasp. J Insect Physiol 50:73–83.

Smith AA, Millar JG, Hanks LM, Suarez AV (2012) Experimental evidence that workers recognize reproductives through cuticular hydrocarbons in the ant *Odontomachus brunneus*. Behav Ecol Sociobiol 66:1267–1276.

Soro A, Ayasse M, Zobel MU, Paxton RJ (2011) Kin discriminators in the eusocial sweat bee *Lasioglossum malachurum*: the reliability of cuticular and Dufour's gland odours. Behav Ecol Sociobiol 65:641–653.

Spiewok S, Schmolz E, Ruther J (2006) Mating system of the European hornet *Vespa crabro*: Male seeking strategies and evidence for the involvement of a sex pheromone. J Chem Ecol 32:2777–2788.

Steiner S, Hermann N, Ruther J (2006) Characterization of a female-produced courtship pheromone in the parasitoid *Nasonia vitripennis*. J Chem Ecol 32:1687–1702.

Steiner S, Mumm R, Ruther J (2007) Courtship Pheromones in parasitic wasps: Comparison of bioactive and inactive hydrocarbon profiles by multivariate statistical methods. J Chem Ecol 33:825–838.

Strohm E et al (2008) A cuckoo in wolves' clothing? Chemical mimicry in a specialized cuckoo wasp of the European beewolf (Hymenoptera, Chrysididae and Crabronidae). Front Zool 5:2.

Sullivan BT (2002) Evidence for a sex pheromone in bark beetle parasitoid *Roptrocerus xylophagorum*. J Chem Ecol 28:1045–1063.

Syvertsen TC, Jackson LL, Blomquist GJ, Vinson SB (1995) Alkadienes mediating courtship in the parasitoid *Cardiochiles nigriceps* (Hymenoptera: Braconidae). J Chem Ecol 21:1971–1989.

Tannure-Nascimento IC et al (2007) Colony membership is reflected by variations in cuticular hydrocarbon profile in a Neotropical paper wasp, *Polistes satan* (Hymenoptera, Vespidae). Genet Mol Res 6:390–396.

Tengo J et al (1991) Species specificity and complexity of dufours gland secretion of bumble bees. Comp Biochem Physiol B 99:641–646.

Tentschert J et al (2001) Chemical profiles, division of labor and social status in *Pachycondyla* queens (Hymenoptera: Formicidae). Naturwissenschaften 88:175–178.

Tentschert J, Bestmann HJ, Heinze J (2002) Cuticular compounds of workers and queens in two Leptothorax ant species — a comparison of results obtained by solvent extraction, solid sampling, and SPME. Chemoecology 12:15–21.

Trabalon M, Plateaux L, Peru L, Bagneres A-G, Hartmann N (2000) Modification of morphological characters and cuticular compounds in worker ants *Leptothorax nylanderi* induced by endoparasites *Anomotaenia brevis*. J Insect Physiol 46:169–178.

Turillazzi S et al (2004) Epicuticular lipids and fertility in primitively social wasps (Hymenoptera Stenogastrinae). Physiol Entomol 29:464–471.

Uboni A, Bagneres A-G, Christides J-P, Lorenzi MC (2012) Cleptoparasites, social parasites and a common host: Chemical insignificance for visiting host nests, chemical mimicry for living in. J Insect Physiol 58:1259–1264.

van Wilgenburg E, Ryan D, Morrison P, Marriott PJ, Elgar MA (2006) Nest- and colony-mate recognition in polydomous colonies of meat ants (*Iridomyrmex purpureus*). Naturwissenschaften 93:309–314.

Vander-Meer RK, Saliwanchik D, Lavine B (1989) Temporal changes in colony cuticular hydrocarbon patterns of *Solenopsis invicta* for nestmate recognition. J Chem Ecol 15:2115–2126.

Vereecken NJ, Mant J, Schiestl FP (2007) Population differentiation in female sex pheromone and male preferences in a solitary bee. Behav Ecol Sociobiol 61:811–821.

Wagner D et al (1998) Task-related differences in the cuticular hydrocarbon composition of harvester ants, *Pogonomyrmex barbatus*. J Chem Ecol 24:2021–2037.

Zanetti P et al (2001) Nestmate recognition in *Parischnogaster striatula* (Hymenoptera Stenogastrinae), visual and olfactory recognition cues. J Insect Physiol 47:1013–1020.
